# Effect of Plasma Fibrinogen Levels on the Risk of Stroke in Patients with Type 2 Diabetes: A Systematic Review

**DOI:** 10.1055/s-0043-1777344

**Published:** 2024-01-30

**Authors:** Nicoline Daugaard, Else-Marie Bladbjerg, Moniek P.M. de Maat, Anna-Marie Bloch Münster

**Affiliations:** 1 Unit for Thrombosis Research, Department of Clinical Biochemistry, Esbjerg Hospital, University Hospital of Southern Denmark, Esbjerg, Denmark; 2 Department of Regional Health Research, University of Southern Denmark, Esbjerg, Denmark; 3 Steno Diabetes Center Odense, Odense University Hospital, Odense, Denmark; 4 Department of Hematology, Erasmus MC, University Medical Center Rotterdam, Rotterdam, The Netherlands

**Keywords:** fibrinogen, type 2 diabetes, ischemic stroke, systematic review

## Abstract

**Aims** In this systematic review, we assessed the literature on the association between fibrinogen levels and stroke in patients with type 2 diabetes (T2D).

**Methods** MEDLINE and Ovid searches of English reports were performed on the relation between fibrinogen, stroke, and T2D in humans. The search was completed on May 4, 2023. Studies were eligible when T2D patients ≥18 years had stroke confirmed by computed tomography or magnetic resonance imaging, plasma fibrinogen was measured, and a relation between fibrinogen and stroke in T2D patients was reported. Screening of reports and extraction of data were done independently by two authors, and study quality was assessed by predefined issues.

**Results** Five studies of different designs were included. Three studies reported on significantly increased fibrinogen levels in T2D patients with stroke compared with T2D patients without stroke. Two studies did not observe a significant association between fibrinogen levels and stroke risk.

**Conclusion** No consistent association was observed between fibrinogen levels and risk of stroke in T2D patients. Due to differences in study design, low sample size, and poorly defined study participants, larger and better-defined studies are needed to elucidate the role of fibrinogen as a stroke risk marker in T2D patients.

## Introduction

Ischemic stroke (IS) is caused by a thrombus in a cerebral artery leading to reduced blood supply to the brain tissue. In IS, the clot can be formed in the brain itself (thrombotic stroke) or away from the brain and carried through the bloodstream to the narrow brain arteries (embolic stroke). The majority of strokes (87%) are due to thrombosis, where the thrombus can be either permanent or transient (transient ischemic stroke [TIA]). The other 13% are hemorrhagic strokes (HSs), caused by bleeding in the brain. 1 Globally, stroke is the second leading cause of death and the third leading cause of death and disability combined. 2

An important risk factor for stroke is diabetes, and patients with diabetes are at more than twice the risk of getting an IS than nondiabetic people. 3 In fact, diabetes increases the risk of a first stroke as well as stroke recurrence. 4 Diabetes affects 10.5% of adults (20–79 years) worldwide and the majority are diagnosed with type 2 diabetes (T2D). 5 The increased risk of stroke and cardiovascular diseases in general among T2D patients is not fully explained by the major risk factors (hypertension, smoking, and hypercholesterolemia). 6 It may also be due to the procoagulant state seen in diabetes patients or dysfunction of the vascular homeostasis. 7 The prevalence of T2D is increasing worldwide and is expected to rise even more in the future. 5 This might be due to increasing body mass index (BMI), physical inactivity, and unhealthy diet. 8 9 10 Understanding the underlying mechanisms may help in predicting, preventing, and treating stroke in this growing group of patients.

A possible mechanism via which diabetes can be associated with risk of stroke may involve fibrinogen, a central protein in the coagulation system, and an independent risk factor for stroke and recurrent stroke in the general population. 11 12 Studies have demonstrated that levels of fibrinogen are increased in T2D. 6 13 14 Understanding the relationship between stroke, diabetes, and fibrinogen is valuable in the prediction and treatment of stroke. Fibrinogen is a 340-kDa glycoprotein and is present in the circulation in a concentration of 2 to 5 g/L, but it can exceed 7 g/L during acute inflammation. 15 When fibrinopeptides are cleaved off by thrombin, fibrin monomers are generated that polymerize into long fibers and ultimately into a fibrin clot. 16 It is already known that T2D affects clot characteristics, that is, increases fiber density and decreases clot permeability and lysis, indicating a more prothrombotic clot when compared with healthy individuals. 17 18 19

Although several studies showed a relationship between increased concentrations of fibrinogen and stroke in the general population, 11 12 only a limited number of studies have focused on the association between fibrinogen and stroke in the growing population of patients with T2D. This systematic review summarizes the results of studies analyzing the association between fibrinogen levels and the risk of stroke (IS, HS, and TIA) in patients with T2D.

## Methods

This systematic review was performed in accordance with the Preferred Reporting Items for Systematic Reviews and Meta-Analyses guidelines. 20 It was registered on PROSPERO (registration number: CRD42021286074), and no review protocol was conducted.

### Article Search

We constructed a search strategy in collaboration with a librarian and used it for the systematic literature search in PubMed (MEDLINE) and Embase (Ovid). The following search string was used: (“Diabetes Mellitus” OR “Diabetes” OR “Diabetes Mellitus”[MESH]) AND (“Fibrinogen”[MESH] OR “Fibrinogen*”) AND (“Cerebrovascular disease*” OR “Cerebrovascular Disorder*” OR “Cerebrovascular Disorders”[MESH] OR “hemorrhagic stroke*” OR “brain hemorrhage*” OR “Intracerebral hemorrhage*” OR “Intracranial hemorrhage*” OR “Subarachnoid hemorrhage*” OR “ischemic stroke” OR “stroke*” OR “Transient cerebral ischemia*” OR “transient brain ischemia*” OR “Transient Ischemic Attack*”).

We excluded MEDLINE journals in the Ovid search, and there was no restriction on year of publication. The last search was completed on May 4, 2023.

### Study Selection

Two researchers (E-M.B. and N.D.) independently screened records in two steps using Covidence systematic review software, Veritas Health Innovation, Melbourne, Australia (available at www.covidence.org ). First, we identified records based on their title and abstract. Next, we assessed the selected reports through full-text screening. Reports were considered eligible when they met the following criteria: involving patients ≥18 years of age with T2D and IS, HS, or TIA. The diagnosis of stroke should be confirmed by computed tomography (CT) or magnetic resonance imaging (MRI), fibrinogen levels should be measured in plasma at any time, and the fibrinogen method must be reported. Finally, the study should report on the relationship between fibrinogen level and stroke in T2D patients. We excluded studies not conducted in humans, studies including only type 1 diabetes patients, reports published in languages other than English, reports with no full texts available, abstracts from congresses, letters, editorials, and study protocols.

The reference lists of included reports were assessed for relevant papers through PubMed on January 13, 2022. If fulfilling the inclusion criteria, the studies were included in the systematic review. Disagreements between the two researchers were solved by consensus or by the referee A-M.B.M. Remaining duplicates in the Ovid search were removed manually or removed by Covidence.

### Data Extraction

Two researchers (E-M.B. and N.D.) collected data, independently of each other, to reduce the risk of bias. The following data were collected: first author, year of publication, country of study, type of study, sample size, type of imaging for stroke diagnosis, type of stroke, method used for fibrinogen measurement, and fibrinogen levels in T2D patients with stroke compared with T2D patients without stroke.

### Quality Assessment

The scientific quality of studies included in a systematic review is of utmost importance for the overall conclusion. Since there are no standardized quality criteria, our expert author team decided upon seven issues of importance that should be evaluated for each of the studies, to clarify areas that might affect the results or the transparency of the studies. The issues were sample size considerations (to exclude type 2 errors), fibrinogen measured by a standard method (to compare fibrinogen concentrations between studies), and reporting of the following: duration of diabetes (affects the risk of stroke), characteristics of diabetes patients, medication, comorbidity (possible confounders), and statistical analysis (to judge the validity of study results). Based on these items, the studies were scored from 0 to 7, and all included studies were analyzed in the systematic review, irrespective of the quality score.

## Results

### Study Selection

**Fig. 1:**
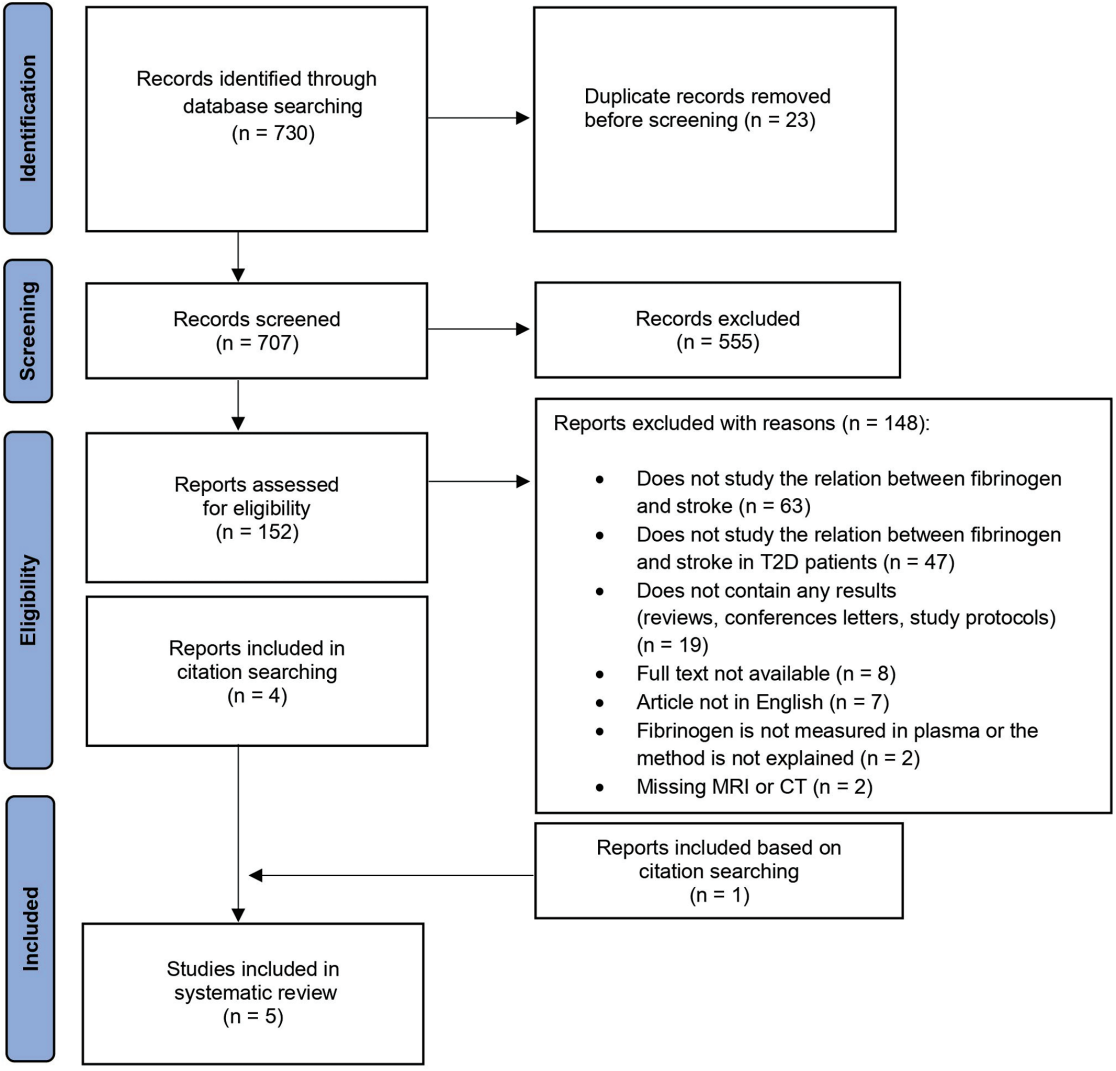
Preferred Reporting Items for Systematic Reviews and Meta-Analyses flowchart showing identification of included reports. CT, computed tomography; MRI, magnetic resonance imaging; T2D, type 2 diabetes.

A total of 730 records were identified through the search. After the selection process, five studies were considered eligible for inclusion ( Fig. 1 ).

### Fibrinogen and Stroke Risk in Type 2 Diabetes Patients

A prospective study 21 measured fibrinogen at study enrolment and followed the patients for the occurrence of stroke for a median of 5 years. They demonstrated a significant increase in fibrinogen levels in T2D patients with stroke compared with T2D patients without stroke and found that fibrinogen levels were positively associated with an increased risk of stroke. Two case–control studies 22 23 showed a significant increase in fibrinogen levels in T2D patients in the nonacute phase at an undefined time point after stroke compared with T2D patients without stroke. Two studies did not observe any significant differences in fibrinogen between T2D patients with or without stroke in either a nested case–control design with >2 years follow-up 24 or a case–control design in which concentrations of fibrinogen were measured in the acute phase (within 24 h) after the stroke. 25 The outcome stroke included TIA or HS in three of the studies, but substudies on stroke type in relation to fibrinogen were not performed. The results are summarized in Table 1 .

**Table 1:** Fibrinogen and stroke risk in type 2 diabetes patients

First author Year of publication, Country of study	Hankey et al 2013 21 Australia, New Zealand, Finland	Asakawa et al 2000 22 Japan	Ali et al 2020 23 India	Bots et al 2002 24 Finland, United Kingdom, The Netherlands	Mistry et al 1990 25 India
Fibrinogen level T2D + stroke T2D − stroke Risk estimate	3.72 (0.81) g/L a c 3.58 (0.74) g/L a HR 1.14 (1.02–1.27)	355.0 (77.1) mg/dL b 326.0 (83.4) mg/dL –	651.1 (123.9) mg/dL c 484.7 (87.3) mg/dL –	– – OR 1.90 (0.98–3.67) a	521.6 (75.2) mg% 478.9 (74.5) mg% –

Abbreviations: HR, hazard ratio; OR, odds ratio; T2D, type 2 diabetes.

“–” indicates that the value was not presented in the study.

Note: Data are expressed as mean and (standard deviation) or (95% confidence interval).

a Baseline levels.

b *p* < 0.05.

c *p* < 0.001.

### Study Characteristics

Characteristics of individual studies are presented in Table 2 . The five studies were published between 1990 and 2020, and they were performed in Asia, Australia/New Zealand, and Europe. The included studies were of different designs: one nested case–control study, one prospective study, and three case–control studies.

**Table 2:** Study characteristics

First author Year of publication Country of study	Hankey et al 2013 21 Australia, New Zealand, Finland	Asakawa et al 2000 22 Japan	Ali et al 2020 23 India	Bots et al 2002 24 Finland, United Kingdom, The Netherlands	Mistry et al 1990 25 India
Type of study	Prospective 5-year follow-up	Case–control	Case–control	Nested case–control	Case–control
Sample size, *N*	Total: 9,795 T2D + stroke: 333 T2D − stroke: 9462	Total: 210 T2D + stroke: 48 T2D − stroke: 162	Total: 186 T2D + stroke: 62 T2D − stroke: 70 − T2D + stroke: 54	Total: 707 T2D ± stroke: 46 − T2D: 661	Total: 96 T2D + stroke: 14 T2D − stroke: 12 − T2D ± stroke: 70
Type of imaging	CT or MRI	CT	CT	Definite stroke: neuroimaging Probable stroke: no imaging	CT
Type of stroke	IS + HS	IS + TIA	IS	IS + HS	IS
Fibrinogen method	Undefined standard assays	Modified clot-rate assay	Electrochemical clot detection method	Clauss (Finland) 42 Nephelometric method (United Kingdom) Prothrombin time assay (The Netherlands) 43	Tyrosine method 44

Abbreviations: CT, computed tomography; HS, hemorrhagic stroke; IS, ischemic stroke; MRI, magnetic resonance imaging; TIA, transient ischemic attack; T2D, type 2 diabetes.

The sample size varied between the studies from 14 to 333 T2D patients with stroke. Three studies used CT as diagnostic tool and two used either CT or MRI. All studies reported on IS. Different methods were used to measure fibrinogen and only two studies 21 24 used a standard method. 26 The time point of fibrinogen measurement in relation to stroke onset differed between the studies. Blood samples were taken within 24 hours after development of stroke symptoms in one study, 25 in the nonacute phase after stroke in two studies, 22 23 and at the baseline examination in two prospective studies. 21 24

### Patient Characteristics

The studies included more men than women, and the age range was comparable between studies. Detailed characteristics of patients with T2D and stroke/no stroke were described in two out of five studies ( Table 3 ). Laboratory data were comparable in these two studies, except for hemoglobin A1c (HbA1c) which was presented in two different units (mmol/mol and percent). In the study of Asakawa et al, 22 patients had T2D for a longer period, a larger percentage of patients were smokers, they had a lower BMI, less patients had hypertension, and more patients had nephropathy or neuropathy compared with the study of Hankey et al. 21 The use of medication in the two studies was not comparable, and within the same study the intake of medication was different between the stroke and nonstroke groups. 21

**Table 3:** Characteristics of patients with type 2 diabetes with and without stroke

Characteristics	Hankey et al 21		Asakawa et al 22		Ali et al 23		Bots et al 24	Mistry et al 25
	Stroke	No stroke	Stroke	No stroke	Stroke	No stroke		
Male, sex (%)	73.6	60.7	54.3 c		71.0	64.5	76.4 d	57.1 d
Age (years)	65.2 (6.2)	61.9 (6.9)	68.4 (7.4)	58.4 (11.0)	[51–80] d		62.7 (10.7) d	57.5 (9.9) d
T2D duration (years)	7 (3–12)	5 (2–9)	9.2 (6.9)	7.0 (6.9)	–		–	–
BMI (kg/m ^2^ )	30.0 (27.2–33.5)	29.8 (26.8–33.6)	24.35 (3.60)	23.43 (3.43)	–		–	–
Smoking (%)	11.4	9.0	25	30.9	–		–	–
Hypertension (%)	73.9	55.4	64.6	41.4	–		–	–
Waist–hip ratio	0.95 (0.07)	0.93 (0.08)	–	–	–		–	–
Atrial fibrillation (%)	5.1	1.7	–	–	–		–	–
HbA1c (mmol/mol a or % b )	54.1 (46.4–65.0) a	50.8 (42.6–61.2) a	7.89 (1.71) b	8.07 (2.20) b	–		–	–
HDL cholesterol (mmol/l)	1.05 (0.23)	1.11 (0.26)	1.15 (0.34)	1.38 (0.39)	–		–	–
Total cholesterol (mmol/l)	5.06 (0.71)	5.03 (0.71)	5.18 (1.04)	5.23 (0.95)	–		–	–
Triglycerides (mmol/l)	1.84 (1.40–2.38)	1.72 (1.34–2.30)	1.63 (1.08)	1.50 (1.02)	–		–	–
Nephropathy (%)	42.0	24.6	47.9	29.6	–		–	–
Neuropathy (%)	24.9	15.9	35.4	29.6	–		–	–
Antihypertensives (%)	77.9	75.4	–	–	–		–	–
Antithrombotics/anticoagulants (%)	48.3	29.1	–	–	–		–	–
Fenofibrate (%)	47.4	50.4	–	–	–		–	–
Antidiabetics (%)			57.2 c		–		–	–
Insulin (%)			25.2 c		–		–	–

Abbreviations: BMI, body mass index; HbA1c, hemoglobin A1c; HDL, high-density lipoprotein; T2D, type 2 diabetes.

Note: Data are expressed as mean (standard deviation), median (interquartile range), or [range].

“–” indicates that the value was not presented in the study.

a mmol/mol

b %

c Indicates that the value was not listed separately for patients with and without stroke.

d Refers to the total group and not only T2D patients.

### Inclusion and Exclusion Criteria

Inclusion criteria in the studies were patients with T2D admitted to the hospital during 1 year 22 or within 24 hours after development of stroke symptoms. 25 Age was also an inclusion criteria with T2D patients aged 50 to 75 years, 21 whereas the study of Bots et al 24 included men aged 42, 48, 54, or 60 years (Finland), men aged 45 to 59 years (United Kingdom), and men and women ≥55 years (The Netherlands). Ali et al 23 included patients based on purposive sampling technique from inpatient and outpatient departments.

The studies had different exclusion criteria, which are summarized in Table 4 . Liver disease(s) was an exclusion criteria in four studies, and renal impairment was an exclusion criteria in three studies. Infections, HS, inflammatory diseases, history of myocardial infarction, and surgery within preceding 3 months were reasons for exclusion in two studies. Most of the exclusion criteria (14/24) came from the study of Ali et al. 23

**Table 4:** Exclusion criteria

	Hankey et al 21	Asakawa et al 22	Ali et al 23	Bots et al 24	Mistry et al 25
Hemorrhagic stroke		✓	✓		
Stroke caused by trauma to the brain, tumor, or infection				✓	
Ischemic cerebral infarction				✓	
Transient ischemic attack				✓	
Subarachnoid or intracerebral hemorrhage				✓	
Liver diseases	✓	✓	✓		✓
Renal impairment	✓		✓		✓
Symptomatic gallbladder disease	✓				
Infections		✓			✓
Inflammatory disease		✓			✓
History of myocardial infarction			✓		✓
Cardiovascular event 3 months before recruitment	✓				
Autoimmune disease		✓			
Nonalcoholic fatty liver disease			✓		
Kidney disease			✓		
Ischemic cardiomyopathy			✓		
Disorders of vessels and blood cells			✓		
Cancer			✓		
Bezafibrate			✓		
β-blockers			✓		
Pentoxifylline			✓		
Ticlopidine			✓		
Surgery within preceding 3 months			✓		✓
Severe dehydration					✓

### Quality Assessment

Seven issues of importance in the quality assessment of the five studies are reported in Table 5 as covered (+) or not covered (−) in the studies. Based on the quality issues defined by us, the overall quality of the included studies was low (three studies scored 0–1 out of 7).

**Table 5:** Quality assessment

	Hankey et al 21	Asakawa et al 22	Ali et al 23	Bots et al 24	Mistry et al 25
Sample size considerations	+	−	−	−	−
Duration of diabetes reported	+	+	−	−	−
Fibrinogen measured by standard method	+	−	−	+	−
Statistical analysis reported	+	+	+	−	−
Medication reported	+	+	−	−	−
Characteristics of diabetes patients reported	+	+	−	−	−
Comorbidity reported	+	+	−	−	−
**Quality score**	**7**	**5**	**1**	**1**	**0**

## Discussion

We identified five relevant articles by screening the literature. These articles fulfilled our inclusion criteria as studies that analyzed the association between fibrinogen and the risk of stroke in patients with T2D. One study observed higher baseline concentrations of fibrinogen in T2D patients who later developed stroke than in T2D patients who did not develop stroke. Two studies reported significantly increased fibrinogen levels in T2D patients in the nonacute phase after stroke compared with T2D patients without stroke. 21 22 23 Two studies did not observe any significant differences in fibrinogen between T2D patients with or without stroke, either prospectively or in the acute phase after stroke. 24 25 To our knowledge, this is the first systematic review investigating the association between fibrinogen and risk of stroke in patients with T2D. This was confirmed by screening the literature and by reviewing the registry for systematic review protocols, PROSPERO.

The largest of the five studies and the one with the highest quality is the study by Hankey et al. 21 To recruit a study population representing the general T2D population, the study had wide inclusion criteria, and T2D patients aged 50 to 75 years were included. Fibrinogen levels were measured in 9,795 T2D patients at enrolment in the study, and the patients were followed for the occurrence of stroke for a median of 5 years. They found that fibrinogen levels were associated with an increased risk of stroke (hazard ratio = 1.14). This risk estimate is lower than the nonsignificant odds ratio of 1.9 in the nested case–control study by Bots et al. 24 This odds ratio was calculated from only 46 T2D patients including an unknown number of stroke cases in a study population characterized by only age and sex. In both prospective studies, fibrinogen levels were measured using a standard method.

Among the case–control studies, the study by Asakawa et al 22 is of highest quality, fulfilling all our criteria. They found significantly increased fibrinogen levels in T2D patients with stroke compared with T2D patients without stroke, results that were confirmed in the case–control study by Ali et al. 23 In both studies, blood samples were obtained in the nonacute phase at undefined time points after stroke. The case–control study by Mistry et al 25 reported on fibrinogen levels in the acute phase after stroke. Measurements of an acute phase reactant in the acute phase of stroke are not comparable with data from studies with measurement in the nonacute phase after stroke. 27 None of the case–control studies used a standard method for fibrinogen measurements, 26 further complicating comparisons between the studies.

A general study limitation is the sample size. Four of the studies included less than 63 patients with stroke and T2D, 22 23 24 25 and sample size might explain the nonsignificant findings of fibrinogen in relation to stroke in the two studies with the fewest T2D patients. 24 25 Only one study considered the size of the study population, 21 even though an appropriate sample size is needed to avoid a statistical risk of type 2 errors, also in subgroups of diabetes patients. Only three of the studies 21 22 23 reported on the statistical analysis, making it difficult to judge the validity of the results for fibrinogen as a risk factor for stroke in T2D patients.

Detailed information about the diabetes patients' characteristics was only given in two studies in which the duration time of T2D was very different. 21 22 The remaining three studies 23 24 25 did not report on duration of diabetes even though increased risk of diabetes-related complications such as atherosclerosis, neuropathy, and nephropathy are well-known, long-term effects of T2D. 28 The long-term disease risk associated with diabetes can be reduced by intensive diabetes control, but HbA1c was only reported in two studies. 21 22

According to the literature, hypertension, and waist–hip ratio are risk factors for stroke, 29 and sex, smoking, age, and ethnicity are risk factors for stroke and affect fibrinogen levels. 6 29 30 31 The sex distribution within the group of stroke/no stroke was only given in two of the studies. 21 23 This may affect the results since fibrinogen levels are higher in women, and the risk of stroke is more frequent in women with T2D than men with T2D. 3 32 There were more smokers among T2D patients with and without stroke in the study of Asakawa et al 22 compared with the study by Hankey et al, 21 and three studies did not report on the distribution of smokers. The ethnicity of the study participants was not presented, however, it is noteworthy that two of the studies reporting a significant increase in fibrinogen levels in stroke patients with T2D were performed in Asia. 22 23 Waist–hip ratio was only reported by Hankey et al, 21 even though the waist–hip ratio is suggested to be a better estimate of visceral fat than BMI. 33 The abovementioned patient characteristics will most likely affect the association between fibrinogen and stroke in T2D patients. This important missing information in three studies 23 24 25 makes it difficult to compare the patient groups and the results from the five included studies.

Among the comorbidities, atrial fibrillation (AF) is important to consider, because AF is the underlying cause in 20 to 39% of stroke cases. 34 35 36 However, AF was only reported by Hankey et al. 21 Medication such as platelet inhibitors and lipid-lowering drugs are known to decrease fibrinogen levels, 31 and antithrombotic medication affects the risk of stroke. 37 Medication is, therefore, a possible confounder in studies where the intake of medication was not evenly distributed between patients with or without stroke, as was the case in Hankey et al, 21 or where the distribution was not reported as in the four remaining studies. 22 23 24 25

Inclusion criteria differed in all studies and included different age groups, men only or both women and men, and time of stroke symptoms. Exclusion criteria also varied, making it difficult to compare the outcome. In some of the studies, exclusion criteria were infections, cancer, or previous cardiovascular events, all known to increase fibrinogen levels 16 38 39 and the risk of stroke, 30 40 41 and intake of antithrombotic medication, which decreases the risk of stroke. 37 These criteria should preferably have been exclusion criteria in all the studies. 16

A limitation in this review is that we might have missed studies in the literature search. However, by carefully selecting the search criteria, searching two databases, and searching the reference lists of included papers, we believe that we have covered a major part of available publications.

In conclusion, we identified five relevant studies of which three observed a significant positive association between fibrinogen levels and risk of stroke in patients with T2D. Due to differences in study design, low sample size, and missing information related to patient characteristics we were not able to clearly identify or reject fibrinogen as a predictor of stroke in T2D patients. Our results demonstrate that there is a clear need for more, larger, and better-defined studies to elucidate the role of fibrinogen as a marker of stroke risk in patients with T2D. If studies of high scientific quality can identify fibrinogen as an independent stroke risk factor in patients with T2D, future studies must address causality and underlying mechanisms, including effects of variants of the fibrinogen molecule. This new knowledge may be a valuable help in predicting, preventing, and treating stroke in the large population of T2D patients.
